# Effectiveness of Psychological Capital Intervention and Its Influence on Work-Related Attitudes: Daily Online Self-Learning Method and Randomized Controlled Trial Design

**DOI:** 10.3390/ijerph17238754

**Published:** 2020-11-25

**Authors:** Shu Da, Yue He, Xichao Zhang

**Affiliations:** Beijing Key Laboratory of Applied Experimental Psychology, National Demonstration Center for Experimental Psychology Education (Beijing Normal University), Faculty of Psychology, Beijing Normal University, Beijing 100875, China; zhuriyinv@163.com (S.D.); hyue0917@163.com (Y.H.)

**Keywords:** psychological capital intervention, online self-learning, job satisfaction, turnover intention, job embeddedness, cost-effectiveness

## Abstract

Research on positive psychology intervention is in its infancy; only a few empirical studies have proved the effectiveness and benefits of psychological capital interventions in workplaces. From a practical perspective, a more convenient intervention approach is needed for when organizations have difficulties in finding qualified trainers. This study aims to extend the psychological capital intervention (PCI) model and examine its influence on work-related attitudes. A daily online self-learning approach and a randomized controlled trial design are utilized. A final sample of 104 full-time employees, recruited online, is randomly divided into three groups to fill in self-report questionnaires immediately before (T1), immediately after (T2), and one week after (T3) the intervention. The results indicate that the intervention is effective at improving psychological capital (PsyCap), increasing job satisfaction, and reducing turnover intention. The practical implications for human resource managers conducting a flexible and low-cost PsyCap intervention in organizations are discussed. Limitations related to sample characteristics, short duration effect, small sample size, and small effect size are also emphasized. Due to these non-negligible drawbacks of the study design, this study should only be considered as a pilot study of daily online self-learning PsyCap intervention research.

## 1. Introduction

In the wake of the influential positive psychology movement, Luthans and Church [1] (p. 59) defined positive organizational behavior (POB) as “the study and application of positively oriented human resource strengths and psychological capacities that can be measured, developed, and effectively managed for performance improvement in today’s workplace”. Instead of emphasizing relatively stable individual differences such as positive personality traits, POB tries to pay more attention to state-like factors that can be developed through well-designed workplace interventions and targeted managerial policies [2].

It is widely assumed that POB can result in highly valued outcomes that benefit employees and organizations [3]. However, compared to the large number of studies that focus on cross-sectional or longitudinal relationships between POB and outcome variables, empirical research focusing on organizational positive psychology interventions is still in its early stages [4]. In the current situation, we do not have enough evidence to support the supposed benefits of positive psychology interventions in organizations, so it will be difficult for human resource (HR) managers to implement positive psychology practices [5], which reveals the huge gap between the requirements from organizations to promote employees’ POB and the tough reality that HR managers do not know how to do so. Therefore, more research on POB interventions is required to not only prove the real value of POBs but also show HR managers how to conduct POB interventions.

Psychological capital (PsyCap), which goes beyond human and social capital, might be one of the most popular POB in both academic and practical fields, and includes four elements: self-efficacy, optimism, hope, and resilience. Many studies have focused on what influences PsyCap and what it leads to [6] but not on PsyCap interventions. Luthans et al. [7] put forward an intervention model, the psychological capital intervention (PCI) model, to operationalize and implement PsyCap interventions. Several intervention studies explore the effectiveness of the PCI model [8,9,10,11]. These interventions are typically conducted by training facilitators using face-to-face or online training, lasting one to three consecutive hours, and utilizing a series of group activities, discussions, or individual exercises [7,9,10]. To sum up, previous PsyCap intervention studies have something in common. First, they are mainly based on the PCI model. Second, they provide one to three hours’ centralized training and do not clarify trainers’ qualifications and styles. Third, they mainly focus on the effectiveness of PsyCap intervention but ignore its influence on other work-related outcomes, except for one study that tests the effect of the intervention on on-the-job performance [9]. Fourth, the participants are all from Western countries.

However, in workplaces, it might be difficult to implement interventions in this way, especially in China. For instance, it is difficult to find a training time suitable for all employees. If employees have emergency work but must attend intervention workshops due to pressure from HR managers, they may be absent-minded or participate halfheartedly, which would counteract the intervention’s effect. In addition, if HR managers make employee participation voluntary, it will cost more money for organizations to make all employees get access to the training. Furthermore, small or middle-sized organizations in small or middle-sized cities in China might face difficulties in finding qualified trainers, as the development of psychological talent is uneven in different regions of mainland China.

Therefore, this study aims to extend the PsyCap intervention literature from both a theoretical and a practical perspective in the cultural context of mainland China. First, we add a goal-as-journey metaphor [12] in the hope of supplementing the PCI model. Second, we utilize a daily online self-learning approach to conduct the intervention. Third, based on the conservation of resources (COR) [13,14] theory, we examine the intervention effect on PsyCap and also other work-related attitudes: job satisfaction, turnover intention, and job embeddedness.

### 1.1. Psychological Capital Development

PsyCap is defined as “an individual’s positive psychological state of development and is characterized by: (1) having confidence (self-efficacy) to take on and put in the necessary effort to succeed at challenging tasks; (2) making a positive attribution (optimism) about succeeding now and in the future; (3) persevering toward goals, and when necessary, redirecting paths to goals (hope) in order to succeed; and (4) when beset by problems and adversity, sustaining and bouncing back and even beyond (resiliency) to attain success” [15] (p. 3). It has been argued that these four elements have a synergistic effect and have more influence when combined than separate [7], which indicates that PsyCap, as a core construct, is better than any of its individual components at predicting outcome variables [16].

Moreover, as the concept of PsyCap is derived from the USA and European countries, it is important to illustrate how we can directly apply the PCI model to non-Western countries. From a theoretical perspective, Lomas [17] introduces the idea of positive cross-cultural psychology and argues that most research actually offers a synthesizing perspective on positive psychology, which recognizes universals in the way well-being is sought, constructed, and experienced, but it also allows for extensive variation in the ways these universals are shaped by culture. However, to the best of our knowledge, the literature on cultural differences in PsyCap interventions is still deficient.

From an empirical perspective, although the concept of PsyCap originates from Western countries, it also attracts wide research interest from many other different countries, such as South Africa [18], Ethiopia [19], Russia [20], Korea [21], Malaysia [22], Pakistan [23], China [24,25,26,27], and so on, which all support the positive effect of PsyCap. A recent meta-analysis [28] suggests that positive psychology interventions that are conducted in non-Western countries have larger effects than those in Western countries. However, research in the field of positive psychology interventions in non-Western countries is still in its infancy, and researchers urge articles from non-Western countries, even when there is a finding of no effect, as this is likely to reduce the publication bias in positive psychology intervention research [28,29].

Therefore, our study aims to generalize the effectiveness of the PCI model in mainland China in order to enrich the literature on positive psychology interventions in non-Western countries. The theory of the development of the four core constructs of PsyCap has been illustrated in detail in the literature [1,7]. Here, we summarize it briefly.

#### 1.1.1. Developing Efficacy

Bandura’s [30] social cognitive theory, especially the content related to efficacy, has been widely accepted. He classified sources of efficacy into four categories: task mastery, modeling or vicarious learning, social persuasion and positive feedback, and physiological or psychological arousal. This became the basic foundation of the PCI model [31]. In the PCI model [7], employees who participate in the intervention will be encouraged to experience and model success through social persuasion and arousal, which will give them a positive emotional experience and confidence to generate plans to achieve their goals. Additionally, combined with hope development, participants can create an imaginary task mastery experience by generating pathways, inventorying resources, and identifying subgoals to enhance and develop their efficacy.

#### 1.1.2. Developing Hope

Positive psychologist Rick Snyder’s work [32] is also the foundation of PsyCap development. He identified the basic components of hope as agency, pathways, and goals. In the PCI model, Luthans and colleagues [7] use a three-pronged strategy, embedded in a goal-oriented framework, which included goal design, pathway generation, and overcoming obstacles. First, employees who participate in the intervention will be guided to form personal goals. Then, they will be asked to divide their goals into several steps. After that, they will be encouraged to think about as many different pathways to the goal as possible. They will also consider the obstacles they might face and figure out alternative solutions to overcome them.

In this study, we add a goal-as-journey metaphor as a prime picture to stimulate participants’ motivation to put their goals into action. Combining the conceptual metaphor theory [33] and identity-based motivation theory [34], researchers have shown that the journey metaphor implies a feeling of knowing how to achieve a goal and promote identity connection [12].

#### 1.1.3. Developing Resilience

The resilience component in the PCI model [7,35,36] mainly comes from Masten [37], who considered asset factors, risks factors, and influence processes as three major components of resiliency. The most effective strategies to build resilience are based on enhancing assets (e.g., becoming more employable) and proactively avoiding risky, potentially adverse events (e.g., meeting critical deadlines) [37]. In the PCI model, employees who participate in the intervention will be encouraged to write down their reactions to recent feedback at work. Then, they will be guided to form a view of reality and how to react with resilience.

#### 1.1.4. Developing Optimism

Luthans and his colleagues [7] developed the optimism dimension of PsyCap from both an expectancy-value orientation and a positive attributional, explanatory style [38], with realistic optimism being the ideal. They proposed that self-efficacy and hope training can both be used to build optimism [7]. Specifically, employees who participate in PCI will be guided to consider “bad events” as potential challenges or obstacles and then use the methods to develop hope to create alternative pathways to overcome problems.

### 1.2. Daily Online Self-Learning Method

In order to widely promote PsyCap interventions in China, HR managers should consider more flexible methods of conducting PsyCap interventions. With the development of the Internet, distance education has gradually become more popular. Increasingly, employees have arranged their spare time to allow for self-learning through distance education. Therefore, online self-learning might replace centralized workshops to implement PsyCap intervention in organizations, and intervention time could be divided into several days to allow employees to autonomously choose an appropriate schedule.

In the literature, self-learning modules are defined as self-contained instructional tools that guide learners through a step-by-step process for achieving educational objectives [39]. It contains some basic elements, including clear objectives and directions, materials needed for accomplishing objectives, and post-tests to examine the effectiveness. The instructional materials usually contain text and images that explain what is to be learnt and include examinations for self-assessment after self-learning. Compared with traditional learning methods with teachers, self-learning is a low-cost, nontechnical, and easily and widely disseminated strategy, which is commonly used by students, nurses, and other groups [40,41,42].

Moreover, traditionally, educators have focused on lecture/discussion teaching methods [43], in which learners are dependent upon instructors and are assumed to have a passive role in learning activities. Lectures assume that all participants are at the same level and learning at the same pace. By contrast, self-learning shows some distinct characteristics. It fosters learners’ personal autonomy, allows them to realize self-management, and lets them control the learning process and utilize an independent approach to learning [44].

To conclude, an online-based self-learning intervention approach enjoys the benefits of speed, convenience, cost, and effectiveness, which can be applied to interventions in workplaces to promote employees’ PsyCap and further human resource development [8]. Additionally, there have been a number of studies examining the effectiveness of online methods to deliver education, training, and interventions [45]. Therefore, we decided to adopt a daily online self-learning method to implement PsyCap intervention and proposed the following hypothesis:

**Hypothesis 1.**  *The daily online self-learning PsyCap intervention will effectively improve the PsyCap level of employees.*

### 1.3. Work-Related Attitudes

In this study, we not only pay attention to the effectiveness of an online self-learning method for promoting PsyCap but also assess the influence of PsyCap interventions on work-related attitudes.

According to psychological resource theories such as the conservation of resources (COR) theory [13,14], individuals are motivated to acquire, maintain, and foster the necessary resources, as found in PsyCap, to attain successful performance outcomes. If an individual builds abundant resources including personal, social, economic, etc., he or she will be more capable of overcoming obstacles, enduring severe stress, and seeing accomplishments [46]. PsyCap, as an underlying capacity consisting of four positive psychological resources, is supposed to be beneficial to employees’ attitudes and performance [47], both in the workplace and in their personal lives, through the mechanism of improving motivation and cognitive processing.

In addition, plenty of empirical studies have already demonstrated the influence of PsyCap on work-related outcomes, such as satisfaction or commitment [48,49], absenteeism [50], and performance [16]. However, except for Luthans et al. [9], who provided evidence that short training interventions can develop participants’ PsyCap and improve on-the-job performance, few intervention studies have attempted to determine whether PsyCap development has a positive influence on employees’ work-related attitudes. In this study, we choose job satisfaction, turnover intention, and job embeddedness as outcome indicators of PsyCap intervention, as they are the most common and fundamental work-related attitudes.

#### 1.3.1. Job Satisfaction

Job satisfaction is a multidimensional construct including cognitive and affective perspectives [51]. From the perspective of cognition, job satisfaction is decided by how much an employee’s physical and psychological needs are fulfilled by his or her work [52]. From the perspective of affect, job satisfaction is the overall feeling toward different aspects of one’s job, including payments, leaders, colleagues, subordinates, environment, content, and so on [53].

With the general expectation of success derived from optimism and the belief in personal abilities derived from efficacy, employees with higher PsyCap show a higher level of job satisfaction, which was also supported by a meta-analysis [54].

#### 1.3.2. Turnover Intention

Employee turnover—employees’ voluntary severance of employment ties [55]—has long attracted the attention of scholars and practitioners [56]. March [57] identifies two main factors that affect employees’ decision to relinquish their job: movement desirability (or job satisfaction) and ease (or perceived job opportunities). Before actual turnover behavior, employees usually generate initial behavior intentions [58] to indicate their possible choices. Avey, Luthans, and Youssef [59] prove that employees’ level of PsyCap has a positive effect on reducing turnover intention as employees with high efficacy are prone to have confidence when facing obstacles (such as high job demands) during work and have higher motivation to overcome difficulties in order to better perform in the organizations; thus, they are more likely to stay within the organization [60].

#### 1.3.3. Job Embeddedness

Mitchell et al. [61] introduce the notion of job embeddedness to elucidate why people stay at an organization or leave. It has been gradually recognized that the motives for leaving and staying are not necessarily opposite to each other [56]. That is, what triggers someone to quit the job (e.g., interpersonal conflicts or low pay) may differ from what makes people want to stay (e.g., opportunities for development or supervisor guidance). Sun et al. [62] prove that, in nurses, high levels of PsyCap are related to higher levels of job embeddedness and performance.

Therefore, we propose the following hypotheses:

**Hypothesis 2.**  *The daily online self-learning PsyCap intervention will effectively promote the job satisfaction of employees.*

**Hypothesis 3.**  *The daily online self-learning PsyCap intervention will effectively reduce the turnover intention of employees.*

**Hypothesis 4.**  *The daily online self-learning PsyCap intervention will effectively promote the job embeddedness of employees.*

## 2. Materials and Methods

### 2.1. Participants

**Figure 1:**
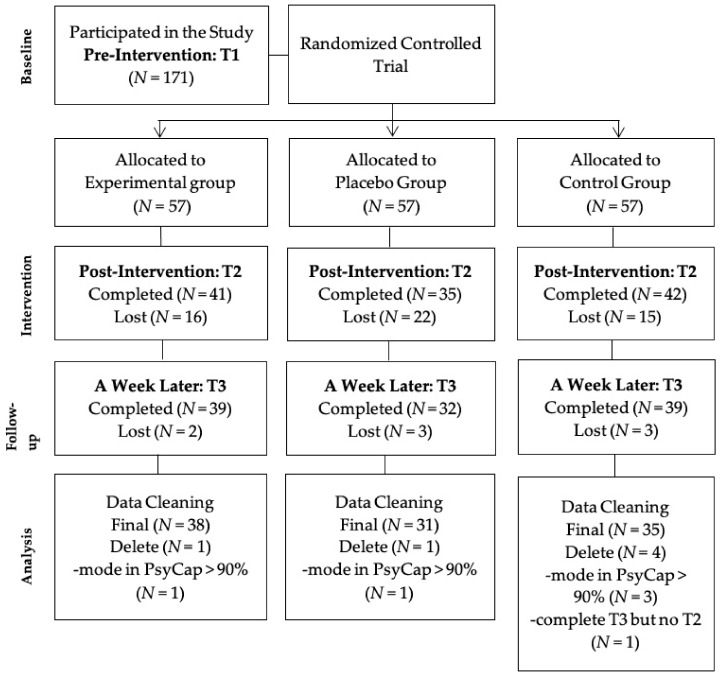
Participants’ flowchart.

Both the recruitment and the intervention in this study were conducted through WeChat, the most popular online social and work connection application in mainland China. The study was open to all who met the following inclusion criteria: 20–60 years old; full-time employees in mainland China; working for five consecutive workdays every week; and ability to access WeChat on a daily basis. Participants were excluded from the study if they had a psychology degree, just in case they were familiar with the procedures of psychology interventions and responded to the surveys in accordance with the purpose of the intervention. We utilized a convenience sampling method, posting the recruitment advertisement on researchers’ WeChat. Due to the snowball effect, we recruited 171 participants at time 1 (T1). Due to dropout, 118 participants completed the questionnaire at time 2 (T2), and 110 participants finished the questionnaire at time 3 (T3). After data cleaning, the final sample consisted of 104 participants (Figure 1). The rate of valid data was 60.82%.

To check for potential selection bias due to dropout, independent-samples *t* tests and chi-square tests were calculated for the study variables and demographic variables. The results showed that the dropouts—those who did not complete post-intervention and follow-up questionnaires—differed with regard to gender, *χ*^2^(1, *N* = 104) = 7.497, *p* < 0.01: more men dropped out of the study than women. The means of the study variables (PsyCap, job satisfaction, turnover intention, and job embeddedness) and other demographic variables (age, job tenure, education, and marital status) did not differ significantly.

The final sample included 38 participants in the experimental group, 31 participants in the placebo group, and 35 participants in the control group; 27 of the total participants were men and 77 were women. Most participants (61.5%) were aged between 26 and 35 years, with 31.7% between 36 and 45, 3.8% between 46 and 55, and 2.9% under 25. As for work experience, 47.1% of the participants had five to 10 years of work experience, 38.5% had more than 10 years, and 14.4% had under five years. Regarding education, 57.7% of the participants had a bachelor’s degree, 33.7% had a Master’s degree, and 8.7% had a high school or associate’s degree. Most participants were married (60.6%), while 34.6% were unmarried, 3.8% were divorced, and 1% were remarried. The sample represented a broad range of industries: the three most common were healthcare (20.0%), education (19.0%), and information technology (9.5%). In addition, the three most common job positions in this sample were consultants (12.4%), human resources personnel (11.4%), and managers (11.4%). The complicated composition of the final sample, the small sample size, and the short follow-up appeared to be limitations of our study and are discussed in detail at the end of the study.

### 2.2. Procedure

All subjects gave their informed consent for inclusion before they participated in the study. The study was conducted in accordance with the Declaration of Helsinki, and the protocol was approved by Institutional Review Board of the Faculty of Psychology, BNU on 19th, December 2019 (201912190084). This study was conducted using a randomized controlled trial (RCT) intervention method.

First, participants voluntarily filled out the informed consent form, baseline questionnaire (T1), and contact information (e-mail address and nickname) online after they viewed the study’s advertisement and decided to participate. We made the study purpose, inclusion and exclusion criteria, and rewards very clear in our online recruitment advertisement. It was stated that this was a three-fold survey on occupational psychological health and the lucky participants could get access to a five-day online activity to promote mental health.

Then, after finishing a pretest questionnaire, participants were randomly assigned to one of three WeChat online groups (assigned by e-mail) according to the sequence in which they finished the pretest: experimental group, placebo group, and control group. After creating three WeChat online groups, the researcher again explained the study purpose and procedures to the different groups. Participants in the control group were thanked for participating in a survey on occupational psychological health and told when and how to complete the online questionnaires three times. As to the experimental and placebo groups, the researcher thanked them for their participation in a survey on occupational psychological health and told them that they were the lucky ones, getting an opportunity for a five-day activity to promote mental health. Therefore, it was a single-blind design, as the participants in the placebo group had the same study guidance but a different intervention from the experimental group to ensure that the results were due to the experimental design instead of the organizational process or instruction. Next, interventions were conducted by sending daily online links to reading materials and practice activities to the experimental group and online links to write down self-reflections to the placebo group for five consecutive workdays. The links were sent in the morning, and participants could choose a convenient time to read and complete the content before the next day. Nothing was sent to the control group. On day six, participants in all three groups were sent a posttest questionnaire (T2). One week later, they were sent a follow-up questionnaire (T3). Participants were asked to complete the intervention in a quiet setting, free from interference. It took approximately 20 min to finish the intervention each day. Each participant won 10-yuan rewards every time they completed a survey.

**Figure 2:**
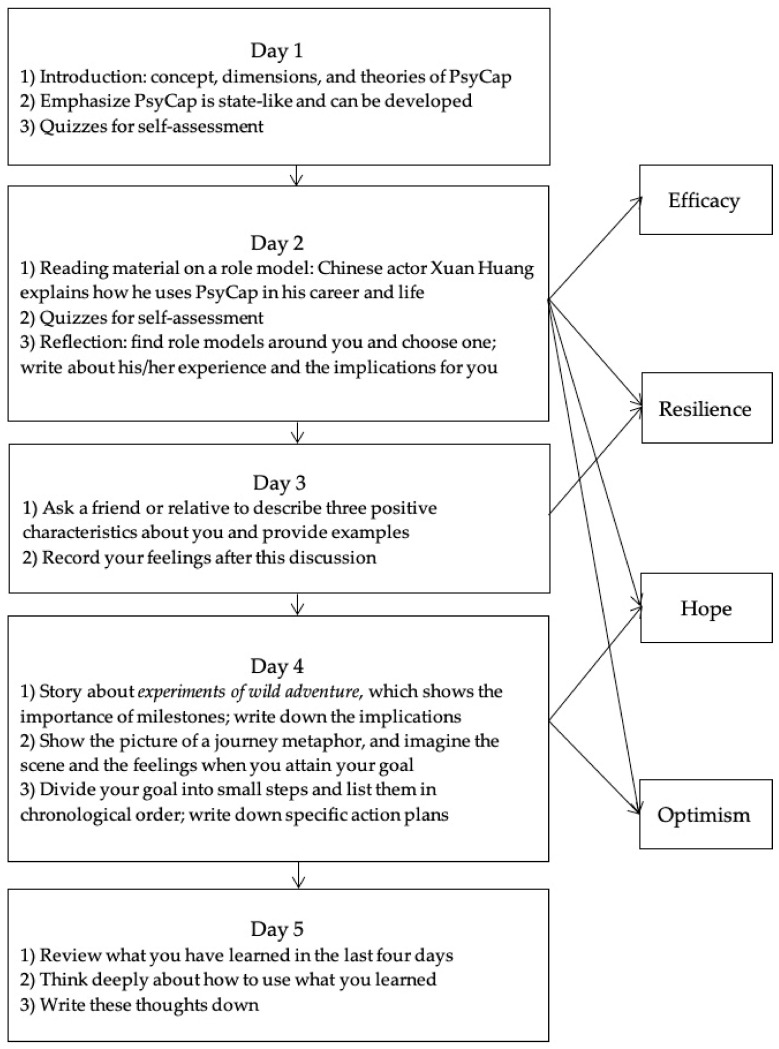
The intervention process.

For PsyCap intervention according to the PCI model and journal metaphor, in the links sent to experimental group, participants were asked to read materials or engage in related activities and record their answers to questions (Figure 2).

In order to balance the Hawthorne effect that may exist in an experimental group, we utilized an expressive writing [63] and a self-reflection [64] intervention method for participants in the placebo group. A growing number of studies have found that expressive writing improves a variety of outcomes, ranging from improved immunity and reduced stress [65]. A meta-analysis [66] reveals that emotional disclosure confers lots of benefits, including increased physical and psychological well-being. Therefore, in the links sent to the placebo group, participants were guided to engage in self-reflection and record anything impressive from the work in at least 50 Chinese characters every day.

### 2.3. Measures

All four study variables (psychological capital, job satisfaction, turnover intention, and job embeddedness) were assessed at all three measurement points (T1, T2, and T3) using the scales outlined below with the exception of demographic variables, which were collected only at T1. All participants created unique nicknames for themselves, which were used to match the three questionnaires. Given that the nicknames might reveal a participant’s identity, we stored them in a separate file. All scales showed good internal consistency at all measurement times (see Table 1).

Psychological capital. Psychological capital was measured by the Psychological Capital Questionnaire-24 developed by Luthans, Avolio, and Norman [16], which included four dimensions: efficacy, optimism, hope, and resilience. All 24 items were rated on a six-point Likert scale, ranging from 1 (*completely disagree*) to 6 (*completely agree*).

Job satisfaction was measured by the six-item Overall Job Satisfaction short form developed by Agho, Price, and Mueller [67]. Items such as “I find real enjoyment in my job” and “I am seldom bored with my job” were rated on a five-point Likert scale, ranging from 1 (*completely disagree*) to 5 (*completely agree*).

Turnover intention was measured with a five-item scale used in previous research by Bluedom [68], including items such as “I intend to quit my present job.” Respondents indicated their answers on a seven-point Likert scale, ranging from 1 (*completely disagree*) to 7 (*completely agree*).

We used the seven-item version of a global measure of job embeddedness [69]. Items such as “I feel attached to this organization” and “It would be easy for me to leave this organization” were accompanied by responses on a five-point Likert scale, ranging from 1 (*completely disagree*) to 5 (*completely agree*).

### 2.4. Data Analysis

SPSS 18.0 and MPLUS 7.0 were used to analyze the data. Descriptive analyses and tests of baseline homogeneity of three groups were conducted using analysis of variance (ANOVA) and chi-square analysis. Data are available from the corresponding author.

To test the hypotheses, we conducted a mixed between-within ANOVA. The interaction of Group (experimental, placebo, control) × Time (T1, T2, T3) was analyzed to test whether three groups showed different development over time. Moreover, we conducted post hoc analyses to test mean differences between three groups at T1, T2, and T3 with three multivariate analyses of variance (MANOVAs). In addition, analysis of covariance (ANCOVA) was used to support the differences between the groups. Then, pairwise comparisons were conducted to test within-group differences, that is, whether the experimental group showed a significant difference in the study variables at different time points. Partial eta-squared (η*_p_*^2^), Cohen’s *d*, and 95% confidence intervals were calculated to examine the effect size. Cohen’s *d* was considered to be a small effect if *d* ≥ 0.2, a medium effect if *d* ≥ 0.5, and a large effect if *d* ≥ 0.8 [70]. Partial eta-squared was interpreted as a small effect if η*_p_*^2^≥ 0.01, a medium effect if η*_p_*^2^ ≥ 0.06, and a large effect if η*_p_*^2^ ≥ 0.14 [70].

## 3. Results

### 3.1. Preliminary Analysis

The ANOVA indicated that random assignment was truly effective at establishing an initial equivalence between the three groups, as no significant differences were found between their levels of PsyCap (*p* = 0.981). Furthermore, at the baseline, no significant differences were found between groups for job satisfaction (*p* = 0.952); turnover intention (*p* = 0.731); job embeddedness (*p* = 0.514) (Table 2); gender, *χ^2^* (1, *N* = 104) = 0.98, *p* = 0.612; age, *χ^2^* (3, *N* = 104) = 4.51, *p* = 0.608; job tenure, *χ^2^* (4, *N* = 104) = 12.81, *p* = 0.118; education, *χ^2^* (3, *N* = 104) = 4.24, *p* = 0.644; or marriage, *χ^2^* (3, *N* = 104) = 3.35, *p* = 0.764.

### 3.2. Intervention Effects on PsyCap

To test the first hypothesis, which proposed that the intervention would effectively improve the PsyCap level, we performed a mixed between-within ANOVA. The results revealed a significant interaction effect for groups by time for PsyCap, *F* = 3.77, *df* = 3.439, *p* = 0.009, η*_p_*^2^ = 0.069. As a post hoc analysis, we conducted MANOVA to test whether the three groups differed significantly regarding their means at T1, T2, and T3 for PsyCap. The results indicated no significant differences between the groups (Table 2).

In addition, we conducted ANCOVA for a more rigorous test for mean differences. Specifically, PsyCap data at T2 and T3 were compared among the experimental, placebo, and control groups, controlling for PsyCap at T1. In addition to controlling for the effect of PsyCap at T1, we also included the covariates of age, gender, job tenure, education, and marriage. The results (Table 3) suggested that the group variable was a significant predictor of PsyCap at T2 and T3 (*p* < 0.05), whereas age, gender, job tenure, education, and marriage were not (*p* > 0.05).

**Figure 3:**
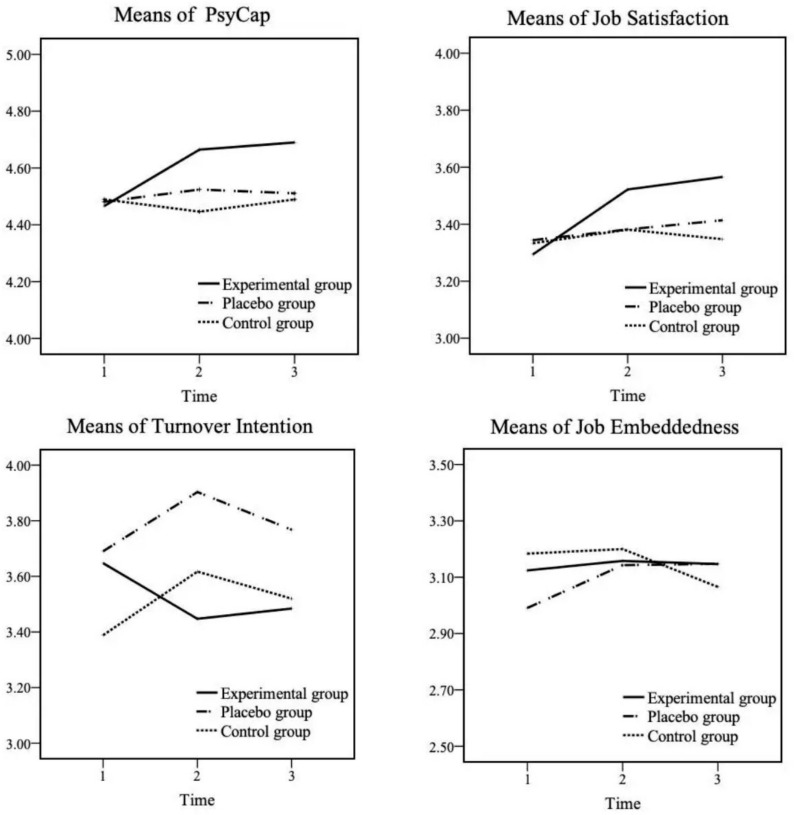
Development of PsyCap and work-related attitudes for the three groups over time.

We also analyzed whether the means in the experimental group were significantly distinct from T1 to T2 and from T1 to T3, expecting an increase across this time frame. Pairwise comparison showed a significant promotion in PsyCap over time in the experimental group (Figure 3): the means changed significantly from T1 to T2 (Δ(T2–T1) = 0.198, *p* = 0.001, *d* = 0.501, 95% CI [0.044, 0.958]) and from T1 to T3 (Δ(T3–T1) = 0.224, *p* = 0.000, *d* = 0.557, 95% CI [0.098, 1.015]). For the placebo group, pairwise comparison showed no significant distinctions from T1 to T2 (Δ(T2–T1) = 0.043, *p* = 1.000, *d* = 0.136, 95% CI [−0.363, 0.634]) or from T1 to T3 (Δ(T3–T1) = 0.03, *p* = 1.000, *d* = 0.098, 95% CI [−0.4, 0.596]). For the control group, pairwise comparison also showed no significant differences from T1 to T2 (Δ(T2–T1) = −0.043, *p* = 1.000, *d* = −0.143, 95% CI [−0.613, 0.326]) or from T1 to T3 (Δ(T3–T1) = 0, *p* = 1.000, *d* = 0, 95% CI [−0.469, 0.469]). Thus, only the experimental group saw a significant promotion in PsyCap level, which supported Hypothesis 1.

### 3.3. Intervention Effects on Work-Related Attitudes

For job satisfaction, the mixed between-within ANOVA showed no significant interaction effect of group by time, *F* = 2.015, *df* = 3.643, *p* = 0.101, η*_p_*^2^ = 0.038. However, the main effect of time was significant, *F* = 4.571, *df* = 1.821, *p* = 0.014, η*_p_*^2^ = 0.043, whereas the main effect of group was not significant, *F* = 0.242, *df* = 2, *p* = 0.786, η*_p_*^2^ = 0.005.

Furthermore, we conducted ANCOVA for a more rigorous test for mean differences of job satisfaction. The results (Table 3) suggested that the group variable was not a significant predictor of job satisfaction at T2 (*p* > 0.05) but was a marginally significant one at T3 (*p* = 0.050), whereas age, gender, job tenure, education, and marriage were not significant at either interval (*p* > 0.05).

In addition, pairwise comparison showed a significant promotion in job satisfaction over time in the experimental group (Figure 3): the means changed significantly from T1 to T2 (Δ(T2–T1) = 0.228, *p* = 0.002, *d* = 0.559, 95% CI (0.1, 1.017)) and from T1 to T3 (Δ(T3–T1) = 0.272, *p* = 0.001, *d* = 0.634, 95% CI (0.173, 1.095)). For the placebo group, pairwise comparison showed no significant differences from T1 to T2 (Δ(T2–T1) = 0.038, *p* = 0.642, *d* = 0.092, 95% CI (−0.406, 0.59)) or between T1 and T3 (Δ(T3–T1) = 0.070, *p* = 0.424, *d* = 0.15, 95% CI (−0.349, 0.648)). For the control group, pairwise comparison showed no significant differences from T1 to T2 (Δ(T2–T1) = 0.048, *p* = 0.531, *d* = 0.099, 95% CI (−0.369, 0.568)) or from T1 to T3 (Δ(T3–T1) = 0.014, *p* = 0.082, *d* = 0.037, 95% CI (−0.431, 0.506)). Thus, the experimental group saw a significant promotion of job satisfaction level, which supported Hypothesis 2.

For turnover intention, the mixed between-within ANOVA showed a marginally significant interaction effect of group by time, *F* = 2.476, *df* = 3.607, *p* = 0.052, η*_p_*^2^ = 0.047. However, neither the main effect of time, *F* = 0.842, *df* = 1.804, *p* = 0.422, η*_p_*^2^ = 0.008, nor the main effect of group, *F* = 0.318, *df* = 2, *p* = 0.728, η*_p_*^2^ = 0.006, was significant.

Nevertheless, the mixed between-within ANOVA revealed a significant interaction effect of group at two time points (T1 and T2), *F* = 4.022, *df* = 2, *p* = 0.021, η*_p_*^2^ = 0.074, which indicated that the effect of the PsyCap intervention on turnover was significant at T2, but the significant effect did not persist to T3. We conducted ANCOVA for a more rigorous test for mean differences of turnover intention; the results shown in Table 3 suggested that the group variable was a significant indicator of turnover intention at T2 (*p* < 0.05) but not at T3 (*p* > 0.05), whereas age, gender, job tenure, education levels, and marriage status were not significant at either interval (*p* > 0.05).

We also analyzed whether the means of turnover intention in the experimental group were significantly changed from T1 to T2, indicating a decrease across this time frame. However, pairwise comparison showed no significant reduction in turnover intention in any of the groups between T1 and T2: the experimental group, Δ(T2–T1) = 0.200, *p* = 0.094, *d* = −0.273, 95% CI (−0.724, 0.179) (Figure 3); the placebo group, Δ(T2–T1) = −0.213, *p* = 0.107, *d* = 0.261, 95% CI (−0.239, 0.761); or control group, Δ(T2–T1) = −0.229, *p* = 0.067, *d* = 0.304, 95% CI (−0.167, 0.776). Although the experimental group did not exhibit a significant reduction in turnover intention level, it appeared that the intervention did prevent participants from increasing turnover intention, which was observed in both the placebo and control groups. Thus, Hypothesis 3 was partially supported.

For job embeddedness, the mixed between-within ANOVA showed a marginally significant interaction effect of group by time, *F* = 2.422, *df* = 4, *p* = 0.050, η*_p_*^2^ = 0.046. However, neither the main effect of time, *F* = 1.794, *df* = 2, *p* = 0.169, η*_p_*^2^ = 0.017, nor main effect of group, *F* = 0.067, *df* = 2, *p* = 0.936, η*_p_*^2^ = 0.001, was significant. Additionally, the ANCOVA results (Table 3) suggested that the group variable was not a significant predictor of job embeddedness at T2 (*p* > 0.05) but was a marginally significant one at T3 (*p* = 0.053), whereas age, gender, job tenure, education, and marriage were not significant at either interval (*p* > 0.05).

In addition, despite expecting an increase over time, pairwise comparison of the means of job embeddedness from T1 to T2 and from T1 to T3 showed no significant promotion in job embeddedness over time in the experimental group (Figure 3): the means did not differ significantly from T1 to T2 (Δ(T2–T1) = 00.034, *p* = 0.572, *d* = 0.167, 95% CI (−0.284, 0.617)) or from T1 to T3 (Δ(T3–T1) = 00.023, *p* = 0.712, *d* = 0.109, 95% CI (−0.341, 0.559)). However, for the placebo group, the pairwise comparison showed significant changes from T1 to T2 (Δ(T2–T1) = 0.152, *p* = 0.023, *d* = 0.327, 95% CI (−0.174, 0.828)) and from T1 to T3 (Δ(T3–T1) = 00.157, *p* = 0.027, *d* = 0.305, 95% CI (−0.196, 0.806)). For the control group, the pairwise comparison showed no significant differences from T1 to T2 (Δ(T2–T1) = 0.016, *p* = 0.794, *d* = 0.058, 95% CI (−0.41, 0.527)) or from T1 to T3 (Δ(T3–T1) = −0.118, *p* = 0.075, *d* = −0.281, 95% CI (−0.752, 0.19)). Thus, the experimental group did not see a significant improvement in job embeddedness. Thus, Hypothesis 4 was not supported.

## 4. Discussion

The purpose of this study was to extend PsyCap intervention studies from both theoretical and practical perspectives in the cultural context of mainland China, which creatively contributes to the academic literature and, to some degree, organizational practice on PsyCap intervention. Theoretically, we added a goal-as-journey metaphor [12] in the development of the hope dimension. To the best of our knowledge, this is the first attempt to extend the existing content of the PCI model. We believe that it is crucial to generalize the effectiveness of PsyCap interventions [7,8,9,10] and at the same time continuously develop previous theories and models. However, it is regrettable that we could not evaluate the effect of adding the journal metaphor into the PCI model. It has been argued by many researchers that the effectiveness of workplace interventions cannot look only at final outcomes [71]. The combination of an outcome evaluation and an evaluation of the process of the intervention is encouraged by researchers [72,73,74].

Moreover, based on the conservation of resources (COR) theory [13,14], we examined not only the effectiveness of this PsyCap intervention but also its influence on other work-related attitudes: job satisfaction, turnover intention, and job embeddedness. Consistent with both our hypothesis and the results of other studies [8,9,10], we found that our intervention based on the PCI model significantly enhanced PsyCap. In addition, we found that our intervention significantly improved the job satisfaction level in the experimental group over time, despite the group difference at the three time points not being significant. Moreover, we found that our intervention significantly reduced the turnover intention at time 2, whereas the intervention effect at time 3 was not significant. Specifically, the group difference was significant at time 2, despite the fact that the time difference was not significant. Even though the effect size of PsyCap intervention’s influence on job satisfaction and turnover intention was not strong, it still showed a positive tendency in the experimental group, which is worth further exploration.

Contrary to our expectations, we found no significant intervention effects for job embeddedness. However, it is surprising that the time effect for the placebo group was significant, which means that job embeddedness significantly improved in the placebo group from T1 to T3. Although we did not verify our hypothesis on job embeddedness, we proved that job embeddedness and turnover intention are not opposite to each other and may be influenced by different factors in workplaces [56]. The adverse effects of job embeddedness have also been found by other researchers. For example, Nd and Feldman [75] note that employees with high levels of embeddedness are associated with declining social capital development, which is presumably because they have already amassed contacts and felt less need to cultivate new ones. The reason why the intervention effect of PsyCap on job embeddedness was not significant may be related to the different emphases of PsyCap and job embeddedness. Specifically, PsyCap may pay more attention to the difficulties of work and help people to deal with or overcome them, while job embeddedness may pay more attention to the positive sides of work. We consider this a very interesting finding and encourage future studies that examine the relationships between PsyCap and job embeddedness, as well as the influence of PsyCap intervention on this variable and also on some other work-related outcomes.

Practically, a daily online self-learning approach was utilized to conduct the intervention, which provides more possible methods for implementing PsyCap interventions in workplaces. As discussed, ascertaining that a proposed training intervention is effective per se, regardless of the person or method delivering it, is meaningful to the intervention itself and also to practice [10]. A self-learning perspective also challenges the traditional mindset of interventions with trainers. With the development of technology, the Internet can be quite a useful and convenient medium for psychological interventions; it is able to reach a broad audience, flexible and time-efficient, cost-effective, and anonymous [76], which can help organizations and employees overcome different kinds of difficulties, such as getting access to professional trainers, gathering employees together at the same time, increasing more active participation, and so on. Our study encourages future researchers and managers to be creative and flexible when designing targeted and suitable interventions for organizations and employees.

Furthermore, our study enriches the literature of PsyCap interventions in non-Western countries. To the best of our knowledge, this study is one of very few PsyCap intervention studies in Asia or China. According to a previous meta-analysis [29], 78% of the randomized controlled trials on the effectiveness of positive psychology interventions are conducted in Western countries. A systematic review [28] also reveals that research in the field of positive psychology intervention in non-Western countries is still in its infancy, and the low quality of the studies from non-Western countries may explain the larger difference in effect sizes. Therefore, our study utilized a rigid randomized controlled trial design to practice PsyCap intervention in China and may contribute to the field of positive psychology interventions by better comparing the cultural differences between Western and non-Western countries.

### 4.1. Limitations and Implications for Future Research

Meanwhile, we admit the following limitations of our study. First, our study sample was not confined to one organization. Participants from various occupations were recruited online using a convenience sampling method and then randomly allocated into three groups. Although this makes the sample more representative, this diversity may have produced differences during the intervention, despite no significant difference being found between three groups at T1. Due to differences in organizational culture, the intervention effect may have been contaminated, and the effect of the PsyCap intervention may have been underestimated. This might explain why Hypotheses 2 and 3 were partially supported: employees’ job attitudes and behaviors may have been influenced by some organizational elements. Future research should consider conducting the interventions in the same organizations to further test the effect of the PsyCap intervention. In addition, conducting interventions in the same organizations would allow for the addition of some group-level or organization-level outcome variables to verify the intervention effect on both groups and organizations. Furthermore, our use of self-report methods to measure all variables may have led to common method bias; an intervention study confined to one organization could include other measurements, such as objective data, peer assessment, and leader assessment, to reduce such bias.

Second, our study tested all four variables just before (T1), just after (T2), and one week after (T3) the intervention. A one-week follow-up is definitely not strong enough to verify the duration effect of the intervention. When designing the study, as participants are all recruited online, it is difficult to maintain connections with participants for a month or several months, which may lead to a higher rate of dropout. Therefore, we chose a one-week follow-up. However, there is no doubt that one-week follow-up is not sufficient to prove the duration effect of the intervention. Therefore, future research on PsyCap intervention should consider test the duration effect through a longer follow-up, such as one month or three months.

Third, the final sample size was quite small, with about 30 to 40 participants in each group. Based on the limited sample size, it should be stressed that this is at best a pilot study and no valid conclusions can be reached. In addition, more men dropped out of our study than women, which produced a final sample composed mostly of women (68.4%), suggesting gender bias. Future research should attempt to enlarge the sample size and balance the gender distribution. Last but not least, the effect size of some of our significant findings is also small, and it may have resulted from the short duration of the intervention, the small sample size, and so on. Future research could extend the intervention duration to several weeks, as researchers have found that long-term training can lead to a higher effect than short-term training [77].

To conclude, this study has some non-negligible drawbacks to the study design, such as the short follow-up and small sample size, which makes the conclusions of this study unconvincing. Therefore, this study should only be considered as a pilot study [78] of PsyCap intervention, and future research is required to further verify the effectiveness of a daily online self-learning PsyCap intervention and its influence on other work-related variables.

### 4.2. Practical Implications

Our results are promising for human resources development and management because we demonstrate that a daily self-learning PsyCap intervention over five working days can promote employees’ PsyCap level, increase their job satisfaction, and decrease their turnover intention to some degree. Our findings encourage managers to flexibly apply the PCI model to meet the needs of their organizations and employees. Our study also indicates that PsyCap intervention can be provided through daily self-learning materials without professional trainers, which allows employees to receive a PsyCap intervention more conveniently and at lower cost.

## 5. Conclusions

This study reveals that a daily online self-learning PsyCap intervention is effective at improving PsyCap levels and has positive influences, increasing job satisfaction and decreasing turnover intention. This is another replication and extension of the PCI model and proves the effectiveness of the model on PsyCap and several work-related attitudes.

## Figures and Tables

**Table 1:** Correlations and reliability of all study variables.

	1	2	3	4	5	6	7	8	9	10	11	12
1. PC T1	(0.89)											
2. PC T2	0.77 **	(0.91)										
3. PC T3	0.75 **	0.89 **	(0.93)									
4. JS T1	0.61 **	0.51 **	0.53 **	(0.83)								
5. JS T2	0.48 **	0.55 **	0.58 **	0.80 **	(0.89)							
6. JS T3	0.49 **	0.57 **	0.63 **	0.77 **	0.89 **	(0.90)						
7. TI T1	−0.30 **	−0.27 **	−0.31 **	−0.60 **	−0.53 **	−0.57 **	(0.94)					
8. TI T2	−0.30 **	−0.29 **	−0.36 **	−0.59 **	−0.61 **	−0.63 **	0.90 **	(0.93)				
9. TI T3	−0.27 **	−0.28 **	−0.34 **	−0.57 **	−0.59 **	−0.62 **	0.90 **	0.95 **	(0.96)			
10. JE T1	0.21 *	0.20 *	0.26 **	0.51 **	0.58 **	0.55 **	−0.62 **	−0.65 **	−0.64 **	(0.81)		
11. JE T2	0.19	0.24 *	0.27 **	0.49 **	0.60 **	0.59 **	−0.58 **	−0.65 **	−0.66 **	0.87 **	(0.87)	
12. JE T3	0.21 *	0.28 **	0.34 **	0.47 **	0.57 **	0.64 **	−0.62 **	−0.68 **	−0.70 **	0.83 **	0.88 **	(0.83)

Note. T1 = Time 1; T2 = Time 2; T3 = Time 3. PC = Psychological capital; JS = Job Satisfaction; TI = Turnover Intention; JE = Job Embeddedness. Coefficient alphas appear in parentheses along the diagonal. * *p* < 0.05. ** *p* < 0.01.

**Table 2:** Means, standard deviations, and results of the multivariate analyses of variance (MANOVAs) for study variables at pretest (time 1), posttest (time 2), and follow-up (time 3), comparing experimental, placebo, and control groups.

Variable	Group	*M* (*SD*)			Results of MANOVA					
		Pre (T1)	Post (T2)	Follow-up (T3)	Pre (T1)		Post (T2)		Follow-up (T3)	
					Univariate *F*, *p*-Value	η*_p_*^2^	Univariate *F*, *p*-Value	η*_p_*^2^	Univariate *F*, *p*-Value	η*_p_*^2^
PsyCap	E	4.47 (0.559)	4.66 (0.503)	4.69 (0.513)	0.020, 0.981	0.000	1.673, 0.193	0.032	1.705, 0.187	0.033
	P	4.48 (0.434)	4.52 (0.489)	4.51 (0.473)						
	C	4.49 (0.514)	4.44 (0.554)	4.49 (0.532)						
Job Satisfaction	E	3.29 (0.651)	3.52 (0.632)	3.57 (0.636)	0.048, 0.952	0.001	0.438, 0.646	0.009	0.852, 0.430	0.017
	P	3.34 (0.689)	3.38 (0.812)	3.41 (0.785)						
	C	3.33 (0.795)	3.38 (0.774)	3.35 (0.782)						
Turnover Intention	E	3.65 (1.64)	3.45 (1.51)	3.48 (1.59)	0.314, 0.731	0.006	0.725, 0.487	0.014	0.308, 0.736	0.006
	P	3.69 (1.80)	3.90 (1.63)	3.77 (1.59)						
	C	3.39 (1.69)	3.62 (1.58)	3.52 (1.59)						
Job Embeddedness	E	3.12 (0.471)	3.16 (0.618)	3.15 (0.535)	0.671, 0.514	0.013	0.051, 0.950	0.001	0.172, 0.842	0.003
	P	2.99 (0.899)	3.14 (0.904)	3.15 (0.861)						
	C	3.18 (0.671)	3.20 (0.763)	3.07 (0.609)						

Note. Group: E = Experimental; P = Placebo; C = Control. *N* (experimental group) = 38; *N* (placebo group) = 31; *N* (control group) = 35. *df* = 2; η*_p_*^2^ = partial eta-squared.

**Table 3:** Analysis of covariance (ANCOVA) controlling for study variables at T1, demographic, and job variables.

Dependent Variable	Variables	*F* Value	*p*-Value	Dependent Variable	Variables	*F* Value	*p*-Value
PsyCap at T2	PsyCap at T1	159.629	<0.001	JS at T2	JS at T1	176.522	<0.001
	Age	0.855	0.357		Age	0.001	0.979
	Gender	0.131	0.719		Gender	0.170	0.681
	Job Tenure	0.144	0.705		Job Tenure	0.221	0.639
	Education	0.589	0.455		Education	0.072	0.790
	Marriage	0.534	0.467		Marriage	0.221	0.639
	Group	4.546	0.013		Group	1.688	0.190
PsyCap at T3	PsyCap at T1	129.698	<0.001	JS at T3	JS T1	140.703	<0.001
	Age	0.028	0.867		Age	0.822	0.367
	Gender	2.132	0.148		Gender	0.018	0.893
	Job Tenure	0.092	0.762		Job Tenure	0.813	0.370
	Education	0.663	0.417		Education	0.298	0.587
	Marriage	0.102	0.750		Marriage	0.095	0.758
	Group	5.018	0.008		Group	3.089	0.050
TI at T2	TI at T1	433.628	<0.001	JE at T2	JE at T1	314.839	<0.001
	Age	0.265	0.608		Age	0.013	0.908
	Gender	2.175	0.144		Gender	0.483	0.489
	Job Tenure	0.125	0.724		Job Tenure	0.398	0.530
	Education	2.470	0.119		Education	0.499	0.482
	Marriage	0.616	0.434		Marriage	0.929	0.338
	Group	5.017	0.008		Group	1.044	0.356
TI at T3	TI at T1	419.907	<0.001	JE at T3	JE at T1	230.827	<0.001
	Age	0.182	0.670		Age	0.037	0.848
	Gender	0.397	0.530		Gender	0.701	0.405
	Job Tenure	0.005	0.943		Job Tenure	0.923	0.339
	Education	1.416	0.237		Education	5.835	0.018
	Marriage	0.044	0.835		Marriage	0.405	0.526
	Group	1.544	0.219		Group	3.028	0.053

Note. T1 = Time 1; T2 = Time 2; T3 = Time 3. JS = Job Satisfaction; TI = Turnover Intention; JE = Job Embeddedness.
